# EHMTI-0224. Occipital nerve stimulation for drug-resistant chronic migraine: increasing experience

**DOI:** 10.1186/1129-2377-15-S1-G5

**Published:** 2014-09-18

**Authors:** PE Bermejo, C del Pozo, E Parodi, J Ahijado, P Rey

**Affiliations:** 1 Neurology, Hospital Puerta de Hierro, Majadahonda, Spain; 2 Pain Unit, Hospital Puerta de Hierro, Majadahonda, Spain

## Introduction

Although some prophylactic medications have been proposed to treat chronic migraine (CM) there are still many refractory patients and other treatments are warranted. Peripheral nerve stimulation (PNS) of the occipital nerves is a potentially promising therapy for CM patients.

## Aim

The aim of this study is to evaluate the efficacy and tolerability of PNS of the occipital nerves for the treatment of refractory CM.

## Methods

Twenty one patients (8 men, 13 women, average age 52.8±12.2) meeting the IHS criteria for refractory CM were enrolled in this study and implanted with a neurostimulation device near the occipital nerves. The primary endpoint was the reduction in Analogical Visual Scale (AVS). Patient satisfaction, migraine frequency, side effects and reasons for discontinuation were also studied. Significance level was set at P<0.05.

## Results

Headache severity according to the AVS was reduced from 8.9±0.7 before PNS to 3.3±2.8 after treatment initiation. There was also a significant difference in reduction of number of headache days and 80% of the patients were satisfied or very satisfied with the procedure. The most common adverse event was persistent implant site pain and only one patient required to be explanted due to inefficacy.

## Conclusions

PNS has been explored as a possible treatment option in selective drug-resistant primary headache disorders and, according to our results, this technique may be effective, safe and well tolerated in treating refractory CM. An increasing experience and a more routine use of these techniques can be forecasted in the near future.

No conflict of interest.

